# A Case of Interstitial Lung Disease-Related Pulmonary Hypertension Successfully Treated with Inhaled Iloprost

**DOI:** 10.3390/life14091068

**Published:** 2024-08-27

**Authors:** Ji Hoon Jang, Hang-Jea Jang, Jae Ha Lee

**Affiliations:** Division of Pulmonology and Critical Care Medicine, Department of Internal Medicine, Inje University Haeundae Paik Hospital, Inje University College of Medicine, Busan 48108, Republic of Korea; saturn80396@gmail.com (J.H.J.); okabango21@gmail.com (H.-J.J.)

**Keywords:** pulmonary hypertension, interstitial lung disease, iloprost

## Abstract

Pulmonary hypertension (PH) associated with interstitial lung disease (ILD) (PH-ILD) significantly worsens clinical symptoms and survival, with no effective treatment available. This case report presents the successful treatment of PH-ILD with inhaled iloprost in a patient with idiopathic pulmonary fibrosis (IPF). The patient, a 68-year-old female, was diagnosed with IPF in 2018 and was maintained on pirfenidone. She experienced stable disease until March 2023, when she developed progressive exertional dyspnea, despite stability indicated by a computed tomography (CT) scan, without progression. Transthoracic echocardiography (TTE) and right heart catheterization (RHC) confirmed PH-ILD with a mean pulmonary artery pressure (mPAP) of 43 mmHg. Due to the ineffectiveness of sildenafil and a CT scan indicating stable IPF, a repeat RHC was performed, which showed a worsening of PH (mPAP 62 mmHg). Consequently, inhaled iloprost, at a dosage of 10 mcg every eight hours, was added to the existing antifibrotic agent. After two months, the patient experienced reduced exertional dyspnea and home oxygen requirements. By the seventh month, pulmonary function tests, the six-minute walk test, and RHC parameters (mPAP 37 mmHg) showed marked improvements. This case suggests that inhaled iloprost may be beneficial for managing PH-ILD. Further research is needed to confirm the efficacy of iloprost in PH-ILD treatment.

## 1. Introduction

Pulmonary hypertension (PH) includes a diverse set of disorders defined by an increased mean pulmonary artery pressure (mPAP) exceeding 20 mmHg, measured at rest [1]. The World Health Organization (WHO) divides PH into five distinct categories, of which Group 3, caused by chronic lung disease, such as chronic obstructive pulmonary disease (COPD), interstitial lung disease (ILD), and obstructive sleep apnea, is the second most common group [2].

PH associated with ILD (PH-ILD) is frequently observed in patients with ILDs, with a prevalence ranging from 3.5% to 15% in the early stages, 30% to 50% in advanced disease, and ultimately 60% to 90% in patients awaiting lung transplantation [3,4,5]. PH-ILD is known to be associated with a worse prognosis through diminished functional capacity, more frequent acute exacerbations, higher supplemental oxygen demands, and reduced quality of life [6,7]. Despite its common occurrence in ILD patients and its clinical significance, PH-ILD has lacked established treatments until recently. The INCREASE trial, led by Waxman et al., showed that inhaled treprostinil improves the six-minute walking distance (6MWD) and reduces the frequency of clinical worsening in PH-ILD [8], leading to its approval by the United States Food and Drug Administration and opening new avenues for treatment. However, in countries, including the Republic of Korea, where inhaled treprostinil has not yet been approved, the treatment of PH-ILD remains limited.

We introduce a case in which PH-ILD was successfully treated with inhaled iloprost, which shares a similar mechanism with treprostinil, and was used owing to constraints in the availability of inhaled treprostinil.

## 2. Case Presentation

The patient was a 68-year-old woman who was diagnosed with idiopathic pulmonary fibrosis (IPF) in January 2018 at a tertiary center in the Republic of Korea following a surgical biopsy via video-assisted thoracoscopic surgery, and commenced treatment with pirfenidone in February 2018. She was on insulin and oral hypoglycemic agents for diabetes, a never-smoker, and no distinct environmental exposures were identified. During the follow-up period, considering the results of the pulmonary function test (PFT), six-minute walk test (6MWT), and high-resolution chest computed tomography (HRCT) scans, her IPF remained stable, without acute exacerbation or disease progression.

However, starting in March 2023, the patient experienced progressive dyspnea on exertion and developed edema in both lower extremities, which led to desaturation and eventual hospitalization. Transthoracic echocardiography (TTE) revealed signs indicative of right heart dysfunction, characterized by increased dimensions of the right atrium (RA) and right ventricle (RV), a reduced tricuspid annular plane systolic excursion (TAPSE) of 11 mm, and an elevated RV–RA pressure gradient of 70.6 mmHg. These observations necessitated the implementation of right heart catheterization (RHC). The results showed an mPAP of 43 mmHg, a pulmonary capillary wedge pressure (PCWP) of 8 mmHg, and a pulmonary vascular resistance (PVR) of 16.59 Wood units (WU). Based on these findings and multidisciplinary discussion involving cardiologists and pulmonologists, the patient was diagnosed with PH-ILD, and was started on sildenafil 20 mg every eight hours. Post-discharge, the patient’s edema showed partial improvement; however, despite home oxygen therapy at 5 L/min, the patient’s exertional dyspnea (WHO functional class Ⅲ) persisted. A follow-up TTE four months later demonstrated a reduction in the patient’s RV–RA pressure gradient to 58.1 mmHg (Table 1).

**Figure 1:**
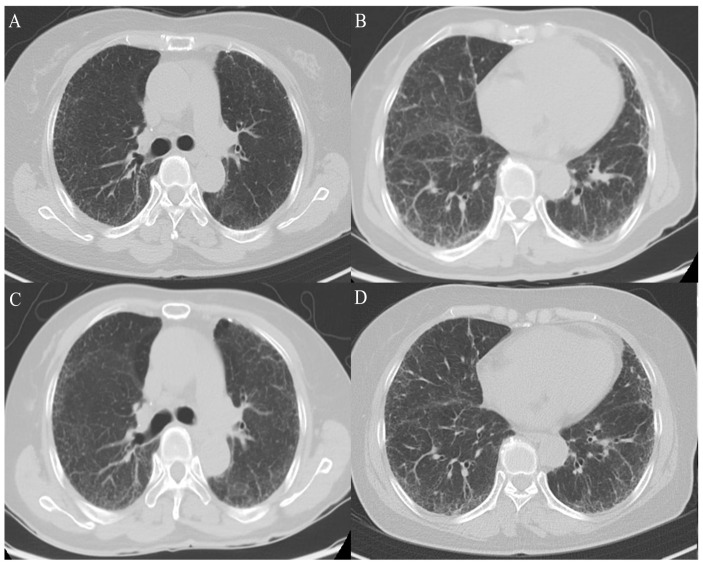
Disease status of IPF over time. A comparison between CT images from 6 October 2022 (**A**,**B**) and 17 October 2023 (**C**,**D**) shows no significant differences. IPF, idiopathic pulmonary fibrosis; CT, computed tomography.

In October 2023, due to ongoing symptoms, the patient was evaluated at the ILD center at Haeundae Paik Hospital in the Republic of Korea. No evidence of pulmonary thromboembolism (PTE) or significant progression of the usual interstitial pneumonia was observed on the chest computed tomography (CT) scan (Figure 1). The PFT revealed a forced vital capacity (FVC) of 2.11 L (69% predicted) and a diffusing capacity for carbon monoxide (DLco) of 3.9 mL/min/mmHg (22% predicted), indicating a disproportionate decline in DLco relative to FVC. Ultimately, due to suspicions of PH-ILD progression, we elected to conduct RHC. A repeat RHC revealed a decreased PVR of 13.78 WU, but an increased mPAP of 62 mmHg and a PCWP of 11 mmHg. We considered administering inhaled treprostinil for PH-ILD. Based on the test results, considering the possibility of a pulmonary vascular phenotype PH-ILD, the patient commenced treatment with inhaled iloprost (Ventavis^®^) at 10 mcg every six hours.

Over the subsequent two months, the dosage of inhaled iloprost was escalated to 20 mcg every six hours, resulting in clinical improvement. The severity of exertional dyspnea improved, with the New York Heart Association class decreasing from 3 to 2, and the WHO functional class decreasing from Ⅲ to Ⅱ. The home oxygen requirement also decreased, allowing a reduction from 5 L/min to 2 L/min. Additionally, a follow-up TTE revealed a decrease in RA and RV dimensions, a reduction in the RV–RA pressure gradient from 72.3 mmHg to 47.3 mmHg, and an increase in TAPSE from 11 mm to 12.8 mm.

**Figure 2:**
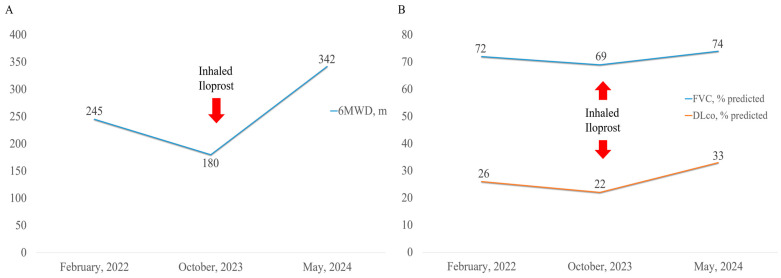
Changes in PFT parameters and 6MWT outcomes following the administration of inhaled iloprost: (**A**) changes in 6MWD and (**B**) changes in FVC and DLco. PFT, pulmonary function test; 6MWT, six-minute walk test; 6MWD, six-minute walking distance; FVC, forced vital capacity; DLco, diffusing capacity of the lungs for carbon monoxide.

**Figure 3:**
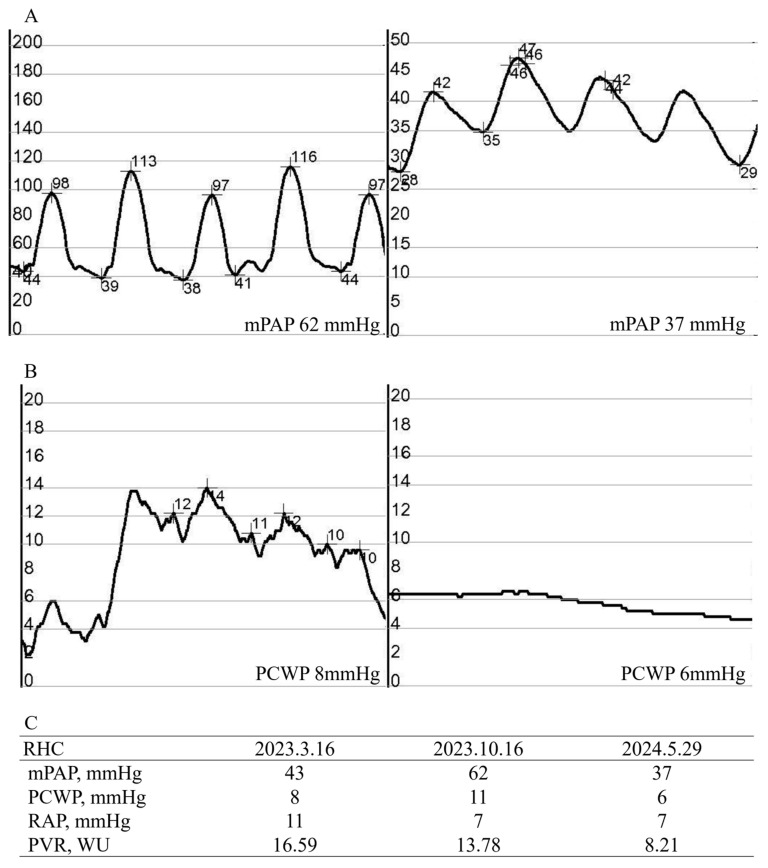
Alterations in RHC parameters following administrations of inhaled iloprost: (**A**) mPAP changes following inhaled iloprost, (**B**) PCWP changes following inhaled iloprost, and (**C**) longitudinal changes in RHC parameters. RHC, right heart catheterization; mPAP, mean pulmonary artery pressure; PCWP, pulmonary capillary wedge pressure; RAP, right atrial pressure; PVR, pulmonary vascular resistance; WU, Wood unit.

Seven months later, a PFT showed an increase in DLco from 3.9 to 5.9 mL/min/mmHg (22% predicted to 33% predicted). The 6MWD improved from 180 m to 342 m, and the nadir oxygen saturation during the test increased from 66% to 73% (Figure 2). Follow-up RHC demonstrated decreases in mPAP to 37 mmHg, PCWP to 6 mmHg, and PVR to 8.21 WU (Figure 3). Significant reductions in mPAP and PCWP, along with PVR, were observed, unlike the previous period when only sildenafil was administered (Figure 3). The patient’s exertional dyspnea improved significantly, allowing for physical activity without home oxygen support, and there was no notable progression of the IPF during the treatment period.

## 3. Discussion

PH-ILD can manifest at any stage of ILD progression and is known to have a high prevalence. However, its underlying mechanisms are not fully understood. Several hypotheses have been proposed, with common factors including hypoxia-induced pulmonary vasoconstriction and the loss of normal lung structure due to inflammatory mediators and fibrosis. These factors contribute to vascular proliferation, remodeling, and fibrogenesis. Most PH-ILD cases exhibit non-severe PH, and severe PH with PVR > 5 WU is relatively uncommon, occurring in 5–10% of cases [2,9]. In PH-ILD, severity does not always directly correlate with the extent and severity of lung fibrosis in ILD progression [10]. In cases where vascular remodeling predominates, referred to as the ‘pulmonary vascular phenotype’, there is typically a more significant decrease in DLco compared to relatively preserved lung function, along with more severe hypoxemia [2,11].

Several agents, including the phosphodiesterase-5 inhibitor sildenafil [12,13,14], endothelin receptor antagonist ambrisentan [15], and the soluble guanylate cyclase stimulator riociguat [16], have undergone clinical trials for the treatment of PH-ILD, but none have shown clear positive effects. However, a notable breakthrough occurred in 2021 during the INCREASE trial utilizing inhaled treprostinil [8]. The prostacyclin analogue treprostinil induced direct vasodilation of the pulmonary and systemic arterial vasculature through pathways mediated by prostaglandin I2 (PGI-2) [17]. The 16-week treatment with inhaled treprostinil resulted in an improved 6MWD, and subsequent study confirmed its long-term clinical efficacy and safety [18]. As inhalation agents preferentially redirect blood flow to the best-ventilated lung areas, the risk of worsening ventilation–perfusion mismatch by systemic vasodilation was reduced [8,19]. Despite the positive results of inhaled treprostinil, many countries are still in the process of approving inhaled treprostinil, precluding its use for patients with PH-ILD. Due to this practical issue, we opted to use inhaled iloprost, which shares a similar mechanism to treprostinil. Iloprost, a prostacyclin mimetic, acts on the PGI-2 receptor to promote vasodilation and anti-proliferative effects similar to treprostinil, but it affects a broader range of platelet receptors, inducing a potent anti-platelet effect [17]. Despite limited research on iloprost, beyond an observational study by Olschewski et al. in 1999 for PH-ILD [20], it provided clinical improvement for our patient. Although the potential synergistic effect of sildenafil and inhaled iloprost on the patient’s improvement should be considered, follow-up studies are needed to evaluate the efficacy of inhaled iloprost monotherapy in PH-ILD patients. Further research is also required to validate its clinical effects and to identify additional treatment options for patients with PH-ILD.

## 4. Conclusions

Our case demonstrates that inhaled iloprost may be considered as an alternative to inhaled treprostinil for patients with PH-ILD exhibiting a pulmonary vascular phenotype. With inhaled treprostinil pending approval in many countries, we anticipate that our case will encourage further research on the clinical effectiveness of inhaled iloprost in PH-ILD patients.

## Figures and Tables

**Table 1:** Longitudinal summary of transthoracic echocardiography findings.

	March 2023	July 2023	October 2023	January 2024
LVEF, %	61	56	58	62
TAPSE, mm	11	11.3	11.8	12.8
TR Vmax, m/s	4.2	3.81	4.25	3.44
PG (RV–RA), mmHg	70.6	58.1	72.3	47.3

LVEF, left ventricular ejection fraction; RV, right ventricular; TAPSE, tricuspid annular plane systolic excursion; TR Vmax, tricuspid regurgitation in systolic jet velocity; PG, pressure gradient; RA, right atrial; IVC, inferior vena cava.

## Data Availability

Original contributions presented in this study are included in this manuscript.
